# Micro-RNA 150-5p predicts overt heart failure in patients with univentricular hearts

**DOI:** 10.1371/journal.pone.0223606

**Published:** 2019-10-10

**Authors:** Masood Abu-Halima, Eckart Meese, Mohamad Ali Saleh, Andreas Keller, Hashim Abdul-Khaliq, Tanja Raedle-Hurst

**Affiliations:** 1 Institute of Human Genetics, Saarland University Medical Center, Homburg/Saar, Germany; 2 Department of Pediatric Cardiology, Saarland University Medical Center, Homburg/Saar, Germany; 3 Center for Clinical Bioinformatics, Saarland University, Saarbruecken, Germany; Institut de Pharmacologie Moleculaire et Cellulaire, FRANCE

## Abstract

**Background:**

In patients with left heart failure, micro-RNAs (miRNAs) have been shown to be of diagnostic and prognostic value. The present study aims to identify those miRNAs in patients with univentricular heart (UVH) disease that may be associated with overt heart failure.

**Methods:**

A large panel of human miRNA arrays were used to determine miRNA expression profiles in the blood of 48 UVH patients and 32 healthy controls. For further selection, the most abundantly expressed miRNA arrays were related to clinical measures of heart failure and selected miRNAs validated by polymerase chain reaction were used for the prediction of overt heart failure and all-cause mortality.

**Results:**

According to microarray analysis, 50 miRNAs were found to be significantly abundant in UVH patients of which miR-150-5p was best related to heart failure parameters. According to ROC analysis, NT-proBNP levels (AUC 0.940, 95% CI 0.873–1.000; p = 0.001), miR-150-5p (AUC 0.905, 95% CI 0.779–1.000; p = 0.001) and a higher NYHA class ≥ III (AUC 0.893, 95% CI 0.713–1.000; p = 0.002) were the 3 most significant predictors of overt heart failure. Using a combined biomarker model, AUC increased to 0.980 indicating an additive value of miR-150-5p. Moreover, in the multivariate analysis, a higher NYHA class ≥ III (p = 0.005) and miR-150-5p (p = 0.006) turned out to be independent predictors of overt heart failure.

**Conclusion:**

In patients with UVH, miR-150-5p is an independent predictor of overt heart failure and thus may be used in the risk assessment of these patients.

## Introduction

Univentricular heart (UVH) disease is a complex and rare congenital cardiac disorder with a functionally single ventricular chamber of right or left ventricular morphology. It accounts for ~7.7% of all congenital heart defects and encompasses a great variety of heart lesions [1]. After several palliative surgical stages resulting in a complete Fontan procedure in early infancy, survival and outcome are mainly dependent on the morphology and function of the major ventricular chamber as well as pulmonary hemodynamics [2, 3]. During long-term follow-up, patients with UVH are prone to develop heart failure, arrhythmias, thromboembolic events and congestive hepatopathy including liver fibrosis, cirrhosis or hepatocellular carcinoma [4–10]. Moreover, all these late complications are strongly associated with worsening of outcome and thus have prognostic impact in this patient population [4, 5].

Studies have demonstrated that microRNAs (miRNAs) play a critical role in the pathogenic mechanisms of heart failure such as remodeling, hypertrophy or apoptosis [11, 12]. Moreover, the etiology but also the different stages of heart failure are associated with differentially expressed miRNA patterns [13–16]. Since extracellular circulating miRNAs are remarkably stable [17], they can be used as diagnostic and prognostic markers for heart failure [16, 18] or to guide response to therapy [19, 20].

In patients with congenital heart disease, miRNAs have been shown to be differentially expressed according to the underlying heart defect [21]. Furthermore, they may also indicate disease progression or the presence of symptomatic heart failure as has been shown previously in patients after surgical repair of tetralogy of Fallot [22]. To date, there is only one study available investigating miRNAs in children with UVH and different stages of palliation in infancy [23]. However, no data are available on the specific miRNAs that are involved in the onset or progression of heart failure in adolescent and adult UVH patients. Therefore, the aim of our study was to analyze miRNAs in the blood of adolescent and adult UVH patients in order to identify those miRNAs that are associated with clinical measures of heart failure and to assess their predictive value for the occurrence of overt heart failure as well as for all-cause mortality in this cohort of patients.

## Materials and methods

### Patients

A total of 48 consecutive UVH patients seen in our outpatient clinic between 02/05/2015 and 18/06/2018 were enrolled in the present study and comprised 32/48 (66.7%) patients with a morphological left and 16/32 (50%) patients with a morphological right ventricle. In the left ventricle UVH group, 12 patients presented with tricuspid atresia, 12 patients with double inlet left ventricle and 8 patients with pulmonary atresia with or without ventricular septal defect. In the right ventricle UVH group, 8 patients had hypoplastic left heart syndrome or mitral atresia and 8 patients double outlet right ventricle with pulmonary stenosis. Mean age was 22.8 ± 10.1 years (range 11–46 years). 17 patients were female and 31 patients male. Patients’ characteristics are illustrated in Table 1.

**Table 1:** Characteristics of UVH patients according to the morphology of the functionally single ventricle.

Variables	All patients (n = 48)	LV morphology (n = 32)	RV morphology (n = 16)	p-value*
Age at follow-up (years)	22.8 ± 10.1	23.6 ± 10.8	21.0 ± 8.6	ns
Patients with incomplete palliation	6/48 (12.5%)	5/32 (15.6%)	1/16 (6.25%)	ns
Patients with overt heart failure	6/48 (12.5%)	4/32 (12.5%)	2/16 (12.5%)	ns
NYHA functional class	1.5 ± 0.7	1.5 ± 0.8	1.6 ± 0.7	ns
Systolic blood pressure (mmHg)	121.4 ± 14.5	123.2 ± 14.9	117.8 ± 13.4	ns
Diastolic blood pressure (mmHg)	66.0 ± 9.9	66.2 ± 10.7	65.6 ± 8.1	ns
Transcutaneous oxygen saturation at rest (%)	91.7 ± 5.3	91.3 ± 5.9	92.3 ± 4.1	ns
Ejection fraction of SV (%)	54.9 ± 6.6	55.3 ± 5.9	54.3 ± 8.1	ns
Enddiastolic volume of SV (ml)	145.6 ± 58.3	133.1 ± 54.7	170.7 ± 58.8	ns
Endsystolic volume of SV (ml)	67.3 ± 31.0	60.9 ± 26.7	80.1 ± 35.7	ns
VTI above aortic valve (cm)	25.4 ± 5.2	25.6 ± 4.6	24.9 ± 6.4	ns
Albumin (g/l)	48.0 (44.0–49.0)	47.5 (44.0–49.0)	48.0 (44.3–49.8)	ns
γGT (U/l)	67.0 (40.5–96.0)	66.0 (39.3–96.0)	67.0 (46.5–106.0)	ns
eGFR (ml/min)	105.9 (85.3–122.5)	102.7 (87.9–117.8)	118.1 (84.0–134.9)	ns
NT-proBNP (pg/ml)	124.3 (60.1–354.2)	147.6 (86.7–354.2)	93.2 (49.9–415.1)	ns
High sensitive troponin T (pg/ml)	4.0 (3.0–6.0)	4.0 (3.0–9.8)	3.5 (3.0–5.8)	ns

UVH, univentricular heart; NYHA, New York Heart Association; LV, left ventricle; RV, right ventricle; SV, systemic ventricle; VTI, velocity time integral; eGFR, estimated glomerular filtration rate; ns, not significant.

Mean ± standard deviation or median (interquartile interval) are used.

* LV compared to RV subgroup.

At enrollment, a structured protocol including a 12-lead surface electrocardiogram, a physical examination, measurement of blood pressure and transcutaneous oxygen saturation at rest, two-dimensional echocardiography as well as a venous blood draw for routine laboratory parameters and blood sampling were performed. The study protocol has been described in detail previously [24]. Follow-up visits were performed annually according to the above mentioned protocol and with special emphasis on the occurrence or presence of overt heart failure (i.e. clinical signs of acute heart failure such as pleural effusions, ascites or peripheral edema associated with significant weight gain) as well as on survival status. Follow-up was extended to 30/06/2019 and comprised a mean time of 35.0 ± 13.8 months.

Thirty-two healthy volunteers served as controls and were matched to UVH patients according to age and gender (S1 Dataset). Control group participants were recruited mainly within our institution and consisted of medical students, medical staff or adolescents admitted for unspecific chest pain. All volunteers underwent physical examination and two-dimensional echocardiography to verify the absence of any heart abnormality as well as venous blood sampling after echocardiography. The study complies with the Declaration of Helsinki, was approved by the Saarland medical association ethical board and all subjects or their guardians gave written and informed consent before enrollment.

### Sample preparation and RNA isolation

In all patients and controls, blood samples for miRNA detection were collected in PAXgene^™^ blood tubes (Becton–Dickinson, Heidelberg, Germany) shortly after echocardiographic evaluation. All PAXgene^™^ blood tubes were stored at room temperature for at least 24 hours to ensure complete lysis of the blood cells, then stored at -20°C for several days and finally transferred to -80°C for long-term storage until RNA isolation. Total RNA including miRNAs was isolated from blood samples using PAXgene^™^ Blood miRNA Kit on the QIAcube^™^ robot (Qiagen, Hilden, Germany) following the manufacturer’s recommendations and included DNase I treatment (Qiagen). To confirm the absence of genomic DNA contamination, a conventional PCR with exon spanning primers for Glyceraldehyde 3-Phosphate Dehydrogenase (GAPDH) was performed. The concentration of isolated total RNA was measured using NanoDrop ND-2000 spectrophotometer (Thermo Fisher Scientific, Massachusetts, United States). RNA purity was estimated by examining the OD 260/280 and the OD 260/230 ratios. The qualities of total RNA were assessed using the Agilent Bioanalyser 2100 Eukaryote Total RNA Nano Series II (Agilent Technologies, California, United States).

### Analysis of miRNAs by microarray

MiRNA abundance analysis was performed on the isolated miRNA fraction in 48 patients with UVH and 32 age- and gender-matched healthy controls using SurePrint^™^8X60K Human v21 miRNA microarrays (Agilent Technologies) according to the manufacturer’s instructions. In total, an input amount of 100 ng of isolated RNA including miRNAs was labeled and subsequently hybridized to the miRNA microarray chip [25]. Subsequently, data were imported into R statistical environment software v.2.14.2 for analysis. Microarray data are available at the Gene Expression Omnibus (www.ncbi.nlm.nih.gov/geo/) under the accession number GSE 136547.

### Analysis of miRNAs by RT-qPCR

Real-time quantitative PCR (RT-qPCR) validation analysis was performed using the StepOnePlus^™^ Real-Time PCR System (Applied Biosystems, Foster City, CA, United States) and the miScript PCR System (Qiagen) according to the manufacturer’s instructions. For miRNA abundance level detection, 250 ng of the total RNA were converted into complementary DNA (cDNA). The resulting cDNA was then diluted to have 0.5 ng/μL input material for miRNA detection. All RT-qPCR experiments were carried out using the Liquid Handling Robot QIAgility^™^ (Qiagen) before performing RT-qPCR. All primer assays used in the current study were provided by Qiagen. Moreover, miRNA reverse transcription control (miRTC) (Qiagen) was performed to assess the performance of the reverse transcription reaction. The melting curve analysis was used to control the specificity of RT-qPCR products. Specificity of amplicons was further confirmed by agarose gel electrophoresis.

### Data analysis

Clinical data of the patients were collected from medical records. Echocardiography was performed using a Vivid^™^ E9 Ultrasound System (GE Healthcare, Horten, Norway). The echocardiographic loops and Doppler images were stored digitally and analysed on an Echopac server (Echopac Version 6, GE Healthcare) as has been described previously [24]. Echocardiographic data sets were assessed by investigators blinded to the laboratory results. Investigators of miRNA signatures were blinded to the clinical, echocardiographic and laboratory data of the patients.

### Statistical analysis

Raw data generated by Agilent Feature Extraction image analysis software was quantile normalized and the differentially abundant miRNAs in patients versus age- and gender- matched healthy control samples were determined using the R statistical environment v.2.14.2 software. A significance level of miRNAs was analyzed by applying an unpaired two-tailed t test, corrected p-value (<0.05, Benjamini-Hochberg False Discovery Rate (FDR) multiple testing correction method), 1.5-fold change cut-off, and area under the receiver operating characteristic curve (AUC) values for each miRNA were computed. For RT-qPCR, the DataAssist^™^ Software v3.0 (Applied Biosystems) was used to calculate the fold-changes in miRNA expression by the equation 2^−ΔCt^ with RNU6B serving as an endogenous control [26].

Clinical data were analysed using standard statistical software (SPSS version 19; SPSS Inc., Chicago, Illinois). Continuous variables are expressed as mean ± standard deviation or median (interquartile interval) as appropriate. Differences between unpaired groups were analysed using a Mann-Whitney-U test for continuous variables and a chi-square test (or Fisher exact test, if numbers were small) for nominal variables. Correlations were evaluated using Spearman’s regression coefficient. For further analysis, biomarker levels were log_10_ transformed due to the skewed distribution of the data. Receiver-operating characteristic (ROC) curve analysis was used for the prediction of overt heart failure and all-cause mortality. Logistic regression analysis was used and implemented into ROC curve analysis in order to evaluate the AUC of combined biomarker models and to assess their potential additive value. Comparison of AUCs was performed using the DeLong method [27]. Multivariate analysis was performed using Cox regression analysis in a stepwise forward manner to identify independent predictors of overt heart failure and all-cause mortality. Variables entered into the multivariate model were those that gave statistically significant results in the univariate analysis. A two-tailed p-value <0.05 was considered statistically significant.

## Results and discussion

### Results

#### Identification of abundant miRNAs

**Fig 1:**
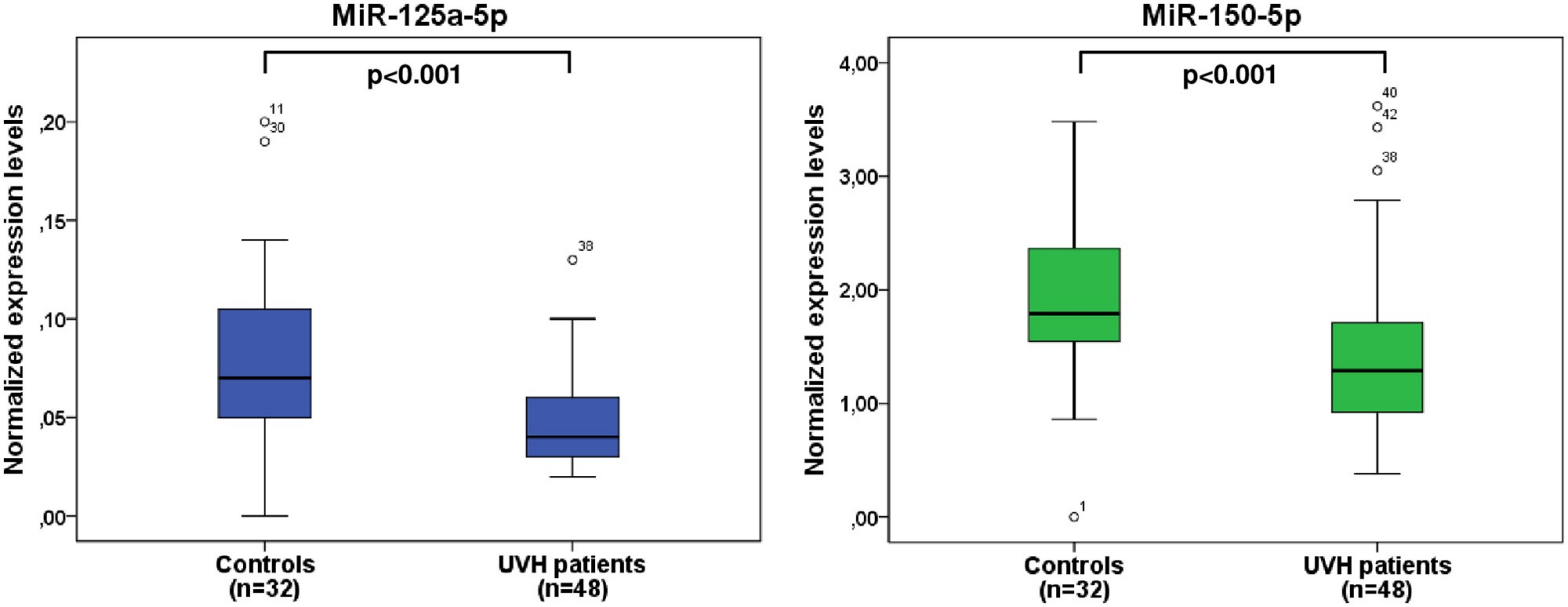
Boxplots displaying normalized expression levels of miR-125a-5p and miR-150-5p in patients with UVH (n = 48) compared to healthy controls (n = 32). UVH, univentricular heart.

To identify miRNAs that were differentially abundant in the blood of patients with UVH and healthy controls, we analyzed abundance level of 2549 human mature miRNAs of miRBase v21. Following background correction and quantile normalization, abundance levels of circulating miRNAs were screened and an unpaired t-test was performed to identify those miRNAs that showed significantly abundant levels in patients with UVH compared to healthy controls. Finally, using Benjamini-Hochberg FDR <0.05 and 1.5 fold change cut-off, 20 down-regulated and 30 up-regulated miRNAs were found in our cohort of patients (S1 Table). Correlation analyses of these abundantly expressed miRNAs to clinical measures of heart failure such as NYHA class, a higher NYHA class ≥ III and the occurrence of overt heart failure were performed identifying miR-125a-5p and miR-150-5p to be the most significant miRNAs. Thus, these two miRNAs were further validated by RT-qPCR indicating significantly lower expression levels in UVH patients compared to healthy controls (Fig 1).

#### Correlation of miRNAs with clinical data

**Fig 2:**
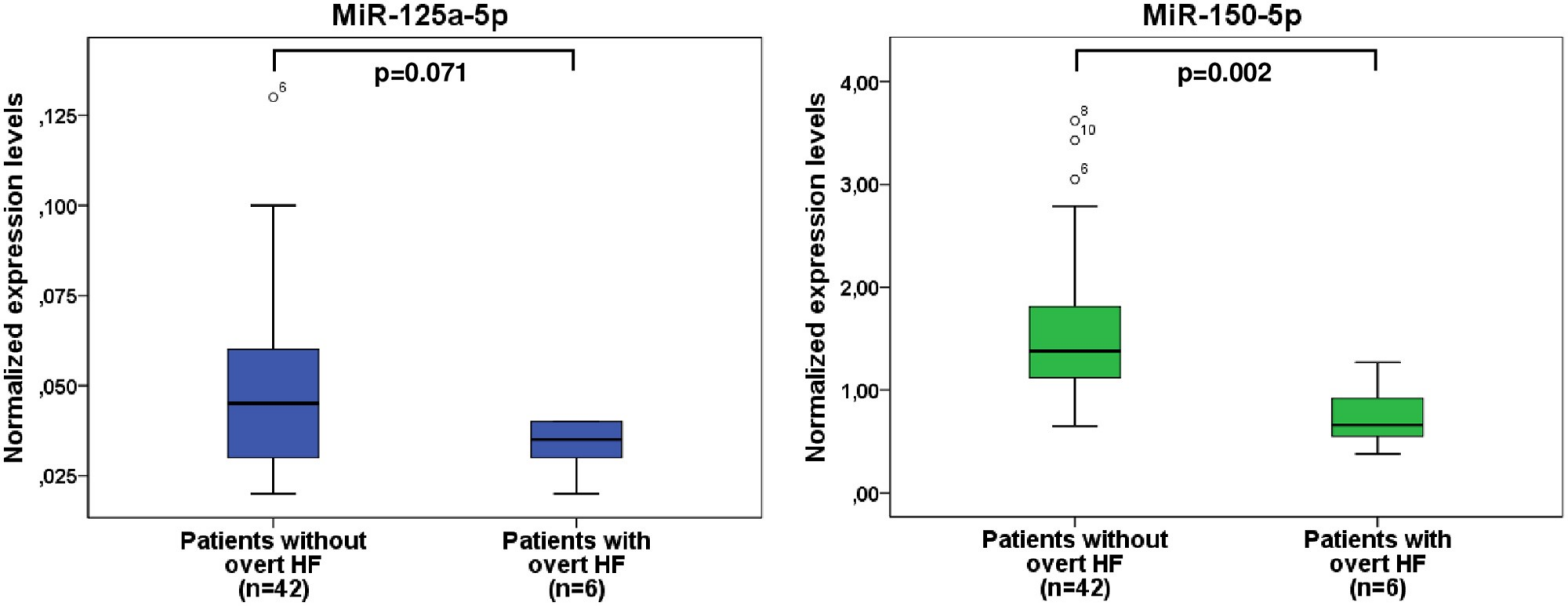
Boxplots illustrating normalized expression levels of miR-125a-5p and miR-150-5p in UVH patients with (n = 6) and without (n = 42) occurrence of overt heart failure. UVH, univentricular heart; HF, heart failure.

During follow-up, overt heart failure requiring intensified medical therapy or rehospitalization occurred in 6/48 (12.5%) patients. In these patients, normalized expression levels of miR-150-5p were significantly lower than in patients without occurrence of overt heart failure (p<0.001) thus indicating significant downregulation of miR-150-5p prior to acute decompensation (Fig 2). Moreover, 6/48 (12.5%) patients died of whom 2 patients due to sudden cardiac death, 3 patients due to progressive heart failure and 1 patient due to septicemia.

Relations of miR-125a-5p and miR-150-5p validated by RT-qPCR to clinical, echocardiographic and laboratory parameters are given in Table 2.

**Table 2:** Correlation of miRNAs validated by RT-qPCR with clinical, echocardiographic and laboratory parameters (n = 48).

	miR-125a-5p		miR-150-5p	
	r	p-value	r	p-value
NYHA functional class	-0.307	0.034	-0.379	0.008
Higher NYHA class ≥ III	-	ns	-0.394	0.006
Occurrence of overt heart failure	-	ns	-0.459	0.001
Death from any cause	-0.298	0.040	-0.382	0.007
Ejection fraction of SV	-	ns	-	ns
VTI above aortic valve	-	ns	-	ns
Albumin	-	ns	-	ns
γGT	-	ns	-	ns
eGFR	-	ns	-	ns
NT-proBNP	-	ns	-	ns
High sensitive troponin T	-	ns	-0.383	0.007

NYHA, New York Heart Association; SV, systemic ventricle; VTI, velocity time integral; eGFR, estimated glomerular filtration rate; ns, not significant.

#### Prediction of overt heart failure

ROC curve analysis was used to identify predictors of overt heart failure in all patients. The most significant predictors were NT-proBNP levels, miR-150-5p, a higher NYHA class ≥ III and high sensitive troponin T levels, respectively. Multivariate analysis identified a higher NYHA class ≥ III (p = 0.005) and miR-150-5p (p = 0.006) as independent predictors of overt heart failure (Table 3).

**Table 3:** Results of ROC curve and multivariate analysis for the prediction of overt heart failure and all-cause mortality.

**Prediction of overt heart failure**				
Variables	AUC	95% CI	p-value	Multivariate analysis (p-value)
NT-proBNP log_10_	0.940	0.873–1.000	0.001	0.322
miR-150-5p	0.905	0.779–1.000	0.001	0.006; HR 19.333 (95% CI 1.553–240.706)
NYHA class ≥ III	0.893	0.713–1.000	0.002	0.005; HR 15.656 (95% CI 1.675–146.369)
High sensitive troponin T log_10_	0.861	0.733–0.990	0.005	0.639
Albumin	0.732	0.435–1.000	0.068	Not included
miR-125a-5p	0.724	0.563–0.886	0.078	Not included
**Prediction of all-cause mortality**				
Variables	AUC	95% CI	p-value	Multivariate analysis (p-value)
NT-proBNP log_10_	0.948	0.887–1.000	<0.001	0.695
High sensitive troponin T log_10_	0.897	0.779–1.000	0.002	0.364
NYHA class ≥ III	0.893	0.713–1.000	0.002	<0.001; HR 36.569 (95% CI 4.255–314.317)
miR-150-5p	0.837	0.693–0.982	0.008	0.084
Albumin	0.778	0.498–1.000	0.029	Not included
miR-125a-5p	0.760	0.590–0.930	0.041	Not included

AUC, area under the curve; CI, confidence interval; HR, hazard ratio; NYHA, New York Heart Association.

**Fig 3:**
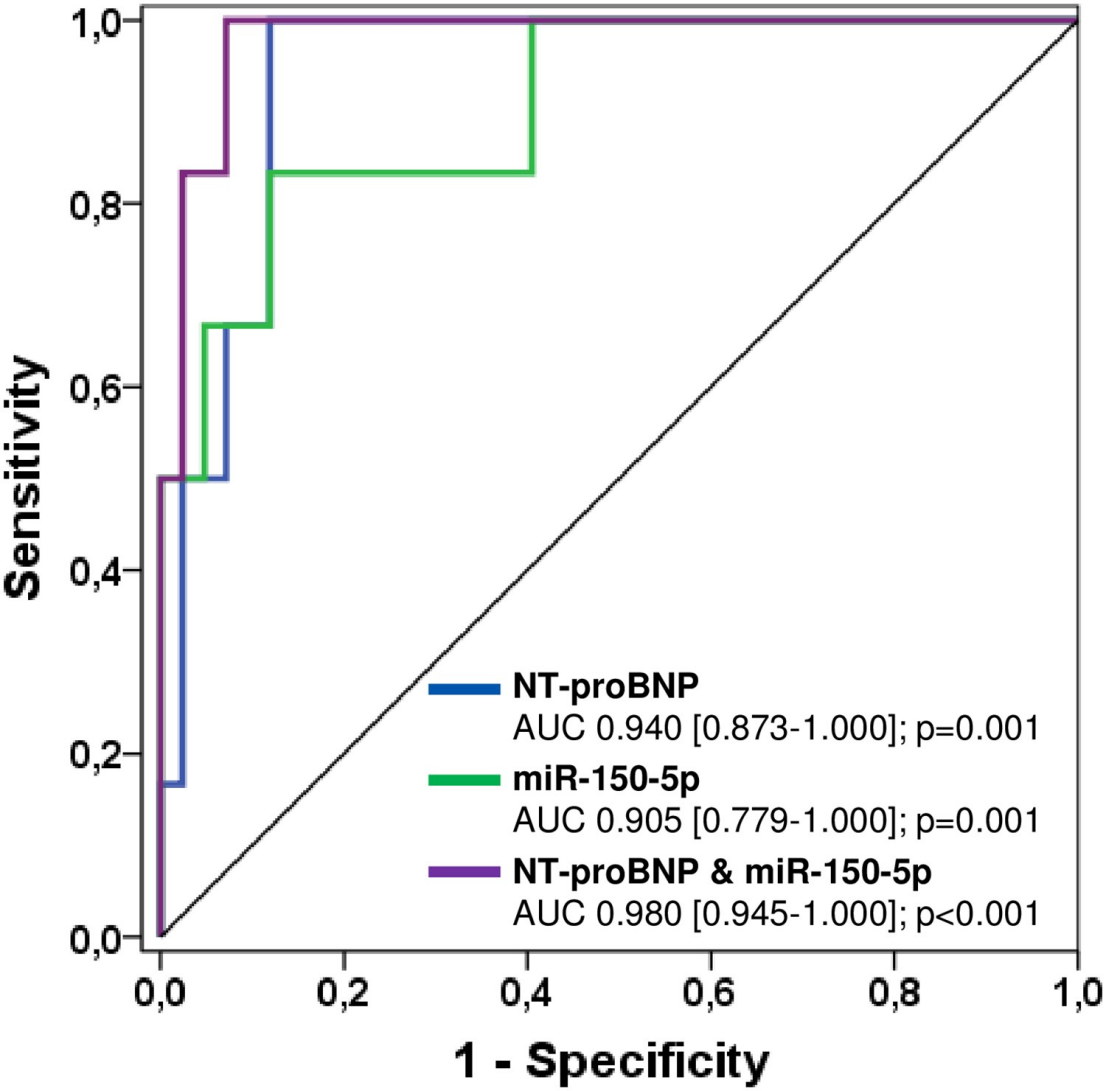
Receiver-operating characteristic (ROC) curves comparing sensitivity and specificity of NT-proBNP and miR-150-5p in predicting overt heart failure. AUC, area under the curve.

The use of a combined model consisting of NT-proBNP and miR-150-5p demonstrated an increase of the AUC to 0.980 (p = 0.116) indicating a potential additive value of miR-150-5p for the prediction of overt heart failure (Fig 3).

#### Prediction of all-cause mortality

**Fig 4:**
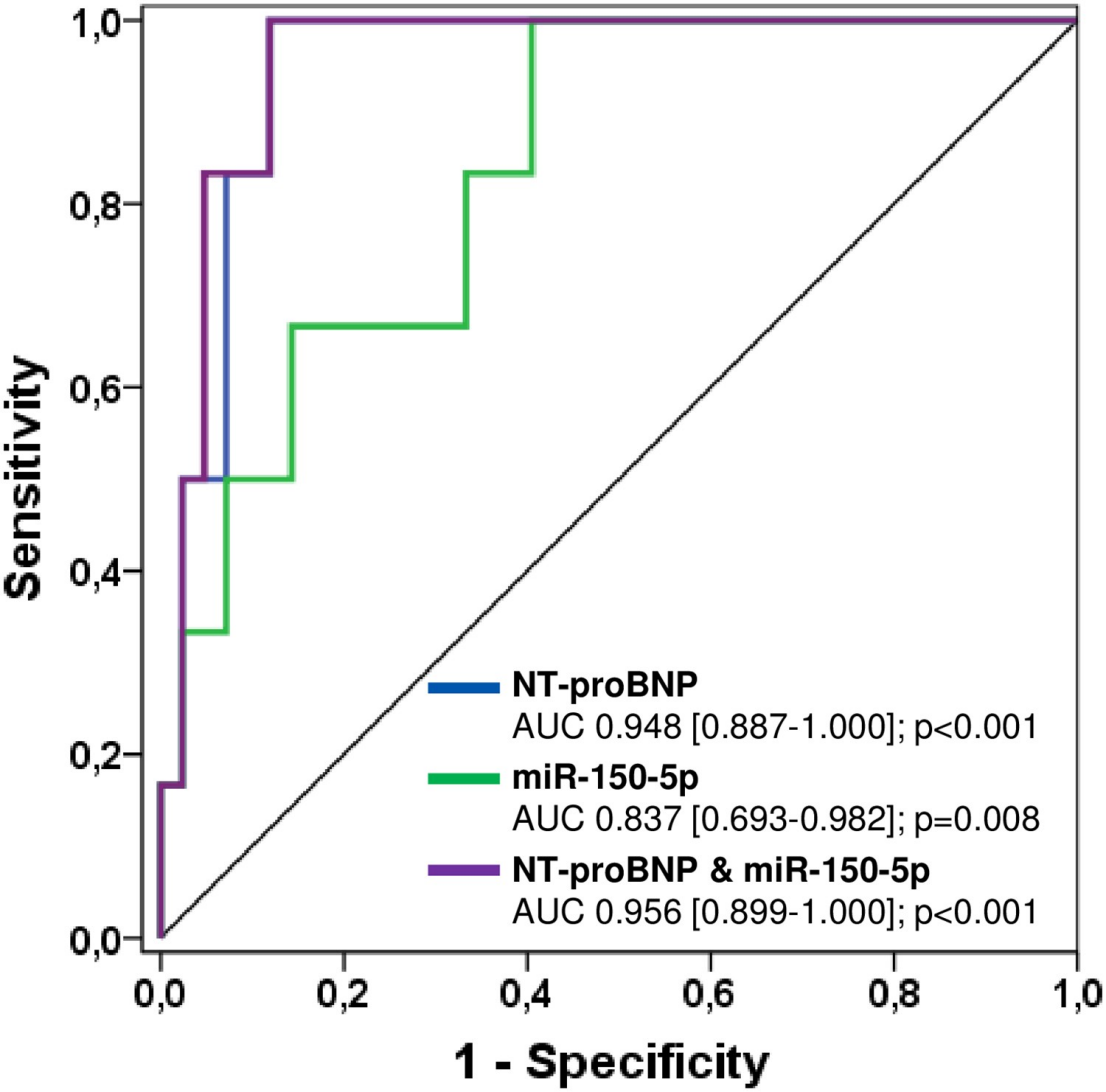
Receiver-operating characteristic (ROC) curves comparing sensitivity and specificity of NT-proBNP and miR-150-5p in predicting all-cause mortality. AUC, area under the curve.

According to ROC curve analysis, the most significant predictors of all-cause mortality were NT-proBNP levels, high sensitive troponin T levels, a higher NYHA class ≥ III and miR-150-5p, respectively. In the multivariate analysis, NYHA class ≥ III (p<0.001) was found to be the most significant independent predictor of all-cause mortality (Table 3). Moreover, AUC only increased slightly to 0.956 using a combined model of NT-proBNP and miR-150-5p (p = 0.675), thus indicating no additive value of miR-150-5p in predicting all-cause mortality (Fig 4).

### Discussion

In patients with left heart failure, miRNAs have been shown to be of diagnostic and prognostic value [16, 18]. Moreover, in patients with tetralogy of Fallot, expression levels of specific miRNAs may indicate the presence of symptomatic heart failure [22]. Thus, the aim of our study was to identify these miRNAs in UVH patients that are significantly associated with clinical measures of heart failure and to assess their predictive value in this cohort of patients.

#### Association of miRNAs with clinical status

In our study, miR-125a-5p and miR-150-5p have been found to be best associated with clinical measures of heart failure in UVH patients using microarray analysis. However, after validation by RT-qPCR, miR-150-5p only turned out to be significantly related to a higher NYHA class or the occurrence of overt heart failure (Table 2). Moreover, miR-150-5p was significantly down-regulated in UVH patients prior to the occurrence of overt heart failure (Fig 2) indicating its pathophysiological relevance of downregulation for heart failure progression or even acute worsening of heart failure. Our results are in line with those found in patients with left heart failure in whom miR-150-5p turned out to be significantly downregulated in those patients with advanced left heart failure or in patients with chronic systolic left heart failure and atrial fibrillation reflecting disease progression and a poor outcome in those patients [28, 29]. Moreover, initial studies have demonstrated that low levels of miR-150-5p predict adverse left ventricular remodeling in patients after acute myocardial infarction [30, 31].

It is of note that miR-150-5p levels were inversely related to measures of heart failure and outcome in our study. Although most of published studies report on positive associations between different circulating miRNAs and outcome, our results are in agreement with previous studies showing a negative association of circulating miR-150-5p with disease severity and outcome in patients with left heart failure [28]. This finding may be due to the fact that miR-150-5p exerts anti-apoptotic functions by directly suppressing distinct pro-apoptotic genes [32] or by inhibiting p53 activity which is a major inducer of apoptosis [33]. As a result, deficiency of miR-150-5p activates apoptosis signaling in cardiomyocytes which is crucial for the progression of heart failure.

In contrast, our results are not in agreement with a study conducted in children with UVH disease in whom miR-129-5p was found to be a sensitive and specific biomarker for heart failure irrespective of ventricular morphology and stage of palliation [23]. However, in that study, a panel of only 5 miRNAs was evaluated not including miR-125a-5p or miR-150-5p. Moreover, the conceptualization of that study did not include a control group and thus was completely different to our study design, which comprised a large panel of miRNAs as well as age- and gender-matched controls in order to select the most differentially expressed miRNAs.

#### Prediction of overt heart failure

The most significant predictors of overt heart failure in our cohort of patients were NT-proBNP levels, miR-150-5p, a higher NYHA class ≥ III and high sensitive troponin T levels with an AUC of 0.940, 0.905, 0.893 and 0.861, respectively. A higher NYHA class ≥ III as well as miR-150-5p turned out to be the strongest independent predictors in the multivariate analysis (p = 0.005 and p = 0.006, respectively). In contrast, echocardiographic measures of heart failure such as ejection fraction of the systemic ventricle or velocity time integral were not predictive of overt heart failure what is not surprising because measurement of these variables is sophisticated due to the heterogeneity of the underlying cardiac malformations and different loading conditions [34]. Moreover, using a combined model of NT-proBNP and miR-150-5p, AUC increased to 0.980 with an AUC difference of NT-proBNP and the combined model of 0.04 (p = 0.116) indicating that miR-150-5p may have potential additive value to NT-proBNP for the prediction of overt heart failure (Fig 3). Overall, our results are in agreement with previous studies demonstrating that downregulation of miR-150-5p seems to play an important role in the progression and deterioration of left heart failure and thus might also yield prognostic impact [28–31].

#### Prediction of all-cause mortality

In our study population, all-cause mortality was best predicted by NT-proBNP levels, high sensitive troponin T levels, a higher NYHA class ≥ III and miR-150-5p with an AUC of 0.948, 0.897, 0.893 and 0.837, respectively. In the multivariate analysis, however, a higher NYHA class ≥ III was the only independent predictor of all-cause mortality (p<0.001). Furthermore, only a slight increase of AUC was seen with the combination of NT-proBNP and miR-150-5p in the ROC curve analysis (Fig 4). Obviously, miR-150-5p seems to be specific for the prediction of overt heart failure but doesn’t add for the prediction of death from any cause. The lack of an additive value for the prediction of all-cause mortality may be due to the fact that mortality was not heart-failure related in 50% of the deceased patients in our study cohort. Nevertheless, natriuretic peptides, troponin T as well as NYHA class are known to be strong predictors of all-cause mortality in patients with congenital heart disease in general but also high-risk subgroups [35, 36].

#### Study limitations

This is the first study that aims to characterize signatures of miRNAs in adolescent and adult patients with UVH using a large panel of more than 2000 miRNAs for initial screening in order to identify those that are involved in the progression of heart failure and might have predictive value in this cohort of patients. Since UVH disease is a rare congenital cardiac disorder, sample size of our patient cohort is rather small and event rate rather low. Especially the number of patients with heart-failure related death is too small to assess the prognostic value of miR-150-5p what would have been most interesting and probably underlined its heart-failure specific profile. Hence, a larger cohort of UVH patients and also a larger control group should be evaluated to provide further insights into the role of miRNAs in these patients.

Another important aspect is the cellular and extracellular origin of miRNAs in our study. Although the ratio of cellular and extracellular miRNA might be different using PAX gene tubes instead of serum or plasma samples, previous studies have shown that disease-associated miRNA signatures originate from the tissues affected by the disease and that extracellular miRNA profiles accurately reflect those signatures found in tissue samples [17, 37, 38]. On the other hand, it is known that expression profiles of extracellular miRNAs may also vary according to the methodology of sample preparation [39] and that normalization methods of extracellular miRNAs have not yet been standardized [38, 40] resulting in inconsistent findings across studies.

## Conclusions

In patients with UVH, miR-150-5p represents an independent predictor of overt heart failure and is significantly down-regulated in patients prior to the occurrence of overt heart failure. Moreover, it seems to have potential additive value to natriuretic peptides for the prediction of overt heart failure and thus may be used as additional biomarker in the risk assessment of these patients.

## Supporting information

S1 Dataset Raw data of patients and controls. (XLSX)

S1 Table Microarray data of the most abundantly expressed miRNAs. (DOCX)
