# Supplementary material for: Micro-RNA 150-5p predicts overt heart failure in patients with univentricular hearts
Source: PLoS One. 2019 Oct 10;14(10):e0223606. doi: 10.1371/journal.pone.0223606 (PMC6786722; doi:10.1371/journal.pone.0223606)
Supplement: S1 Table — (DOCX) [file pone.0223606.s002.docx]

**S1 Table.** Significantly abundant miRNAs in the blood of UVH patients (n=48) compared to age and gender-matched healthy controls (n=32) as determined by microarray analysis (unpaired two-tailed t-test, >1.5-fold difference and Benjamini-Hochberg FDR ≤ 0.05).

| **Micro-RNA** | **Median controls** | **Median patients** | **Log difference** | **Fold change** | **Regulation** | **p-value** | **Corrected p-value** | **AUC** |
| --- | --- | --- | --- | --- | --- | --- | --- | --- |
| miR-101-3p | 2,83 | 5,24 | -2,41 | 5,33 | UP | 1,43E-05 | 0,00228 | 0,69 |
| miR-144-5p | 4,03 | 6,44 | -2,40 | 5,29 | UP | 7,84E-05 | 0,00768 | 0,70 |
| miR-18a-5p | 2,36 | 4,43 | -2,07 | 4,20 | UP | 2,12E-07 | 0,00011 | 0,74 |
| miR-15a-5p | 7,62 | 9,64 | -2,02 | 4,06 | UP | 0,00115 | 0,03446 | 0,69 |
| miR-17-3p | 2,98 | 4,97 | -1,98 | 3,96 | UP | 2,22E-07 | 0,00011 | 0,75 |
| miR-96-5p | 3,85 | 5,81 | -1,96 | 3,89 | UP | 0,00021 | 0,01321 | 0,67 |
| miR-18b-5p | 2,34 | 4,25 | -1,92 | 3,78 | UP | 4,97E-09 | 1,21E-05 | 0,80 |
| miR-144-3p | 3,82 | 5,72 | -1,91 | 3,75 | UP | 0,00028 | 0,01641 | 0,67 |
| miR-17-5p | 6,80 | 8,63 | -1,83 | 3,56 | UP | 0,00064 | 0,02460 | 0,69 |
| miR-20b-5p | 5,97 | 7,54 | -1,57 | 2,96 | UP | 0,00125 | 0,03615 | 0,67 |
| miR-20a-5p | 6,68 | 8,12 | -1,44 | 2,72 | UP | 0,00014 | 0,01147 | 0,68 |
| let-7i-5p | 9,03 | 10,47 | -1,44 | 2,71 | UP | 0,00056 | 0,02333 | 0,69 |
| miR-107 | 9,92 | 11,27 | -1,34 | 2,54 | UP | 0,00016 | 0,01161 | 0,72 |
| miR-103a-3p | 9,64 | 10,79 | -1,15 | 2,23 | UP | 0,00015 | 0,01161 | 0,72 |
| miR-106b-5p | 9,00 | 10,14 | -1,15 | 2,21 | UP | 1,52E-06 | 0,00039 | 0,78 |
| miR-140-5p | 2,78 | 3,90 | -1,12 | 2,17 | UP | 4,32E-05 | 0,00525 | 0,65 |
| miR-1231 | -2,17 | -1,11 | -1,06 | 2,08 | UP | 0,00213 | 0,04644 | 0,69 |
| let-7g-5p | 8,47 | 9,52 | -1,05 | 2,07 | UP | 0,00069 | 0,02548 | 0,68 |
| miR-7-5p | 4,02 | 5,05 | -1,03 | 2,04 | UP | 1,92E-05 | 0,00258 | 0,76 |
| miR-590-5p | 2,27 | 3,22 | -0,95 | 1,94 | UP | 9,05E-06 | 0,00154 | 0,75 |
| miR-301a-3p | 1,79 | 2,73 | -0,94 | 1,91 | UP | 2,80E-06 | 0,00060 | 0,69 |
| let-7f-5p | 9,45 | 10,37 | -0,92 | 1,89 | UP | 0,00112 | 0,03411 | 0,69 |
| miR-148a-3p | 4,63 | 5,53 | -0,90 | 1,87 | UP | 6,31E-05 | 0,00671 | 0,76 |
| miR-454-3p | 3,24 | 4,13 | -0,89 | 1,85 | UP | 0,00110 | 0,03411 | 0,63 |
| let-7a-5p | 10,94 | 11,73 | -0,80 | 1,74 | UP | 0,00201 | 0,04565 | 0,68 |
| miR-29b-3p | 4,32 | 5,11 | -0,79 | 1,73 | UP | 9,04E-06 | 0,00154 | 0,80 |
| miR-29c-3p | 6,26 | 7,04 | -0,78 | 1,71 | UP | 1,83E-06 | 0,00042 | 0,85 |
| miR-19a-3p | 6,63 | 7,40 | -0,77 | 1,71 | UP | 0,00126 | 0,03615 | 0,71 |
| miR-4737 | 1,12 | 1,80 | -0,68 | 1,60 | UP | 0,00196 | 0,04565 | 0,71 |
| miR-210-3p | 6,59 | 7,19 | -0,60 | 1,51 | UP | 1,66E-05 | 0,00249 | 0,83 |
| **Micro-RNA** | **Median controls** | **Median patients** | **Log difference** | **Fold change** | **Regulation** | **p-value** | **Corrected p-value** | **AUC** |
| miR-99b-5p | 4,11 | 3,06 | 1,05 | 2,07 | Down | 8,84E-07 | 0,00028 | 0,21 |
| miR-125a-5p | 5,64 | 4,59 | 1,05 | 2,06 | Down | 4,65E-07 | 0,00020 | 0,18 |
| miR-494-3p | 6,86 | 5,88 | 0,98 | 1,97 | Down | 0,00039 | 0,01960 | 0,25 |
| miR-150-5p | 11,81 | 10,85 | 0,96 | 1,94 | Down | 3,21E-08 | 2,73E-05 | 0,16 |
| miR-199a-5p | 6,04 | 5,10 | 0,94 | 1,92 | Down | 0,00153 | 0,03991 | 0,27 |
| miR-342-3p | 9,77 | 8,89 | 0,87 | 1,83 | Down | 9,46E-09 | 1,21E-05 | 0,12 |
| miR-145-5p | 5,18 | 4,37 | 0,81 | 1,75 | Down | 1,87E-05 | 0,00258 | 0,23 |
| miR-151a-3p | 7,51 | 6,70 | 0,81 | 1,75 | Down | 0,00143 | 0,03912 | 0,29 |
| miR-4485-5p | 5,24 | 4,45 | 0,79 | 1,73 | Down | 0,00051 | 0,02304 | 0,27 |
| miR-6794-3p | 2,94 | 2,17 | 0,77 | 1,71 | Down | 0,00018 | 0,01190 | 0,26 |
| miR-15b-3p | 3,94 | 3,17 | 0,77 | 1,71 | Down | 0,00173 | 0,04271 | 0,29 |
| miR-484 | 9,80 | 9,10 | 0,71 | 1,63 | Down | 0,00032 | 0,01772 | 0,30 |
| miR-7-1-3p | 3,59 | 2,90 | 0,70 | 1,62 | Down | 0,00200 | 0,04565 | 0,29 |
| miR-532-3p | 9,13 | 8,44 | 0,69 | 1,61 | Down | 0,00020 | 0,01321 | 0,23 |
| miR-574-3p | 6,13 | 5,44 | 0,68 | 1,61 | Down | 0,00168 | 0,04250 | 0,24 |
| miR-93-3p | 5,91 | 5,24 | 0,66 | 1,58 | Down | 0,00088 | 0,03001 | 0,19 |
| miR-30b-5p | 11,66 | 11,01 | 0,65 | 1,57 | Down | 0,00235 | 0,04887 | 0,31 |
| miR-23a-3p | 9,42 | 8,82 | 0,60 | 1,51 | Down | 6,77E-05 | 0,00690 | 0,20 |
| miR-324-3p | 9,96 | 9,38 | 0,58 | 1,50 | Down | 7,58E-06 | 0,00149 | 0,24 |
| miR-361-5p | 6,26 | 5,68 | 0,58 | 1,50 | Down | 5,85E-05 | 0,00671 | 0,15 |

AUC, area under the receiver operating characteristic curve.
